# FLOT1 promotes gastric cancer progression and metastasis through BCAR1/ERK signaling

**DOI:** 10.7150/ijbs.82606

**Published:** 2023-10-02

**Authors:** Ran Wang, Wei Huang, Kaimei Cai, Shihan Xiao, Wuming Zhang, Xianqin Hu, Jianping Guo, Linfeng Mao, Weijie Yuan, Yi Xu, Zihua Chen, Zhikang Chen, Chen Lai

**Affiliations:** 1 Department of General Surgery, Xiangya Hospital, Central South University, Changsha,410008, Hunan Province, China.; 2 Hunan Key Laboratory of Precise Diagnosis and Treatment of Gastrointestinal Tumor, Xiangya Hospital, Central South University, Changsha,410008, Hunan Province, China.; 3 National Clinical Research Center for Geriatric Disorders, Xiangya Hospital, Central South University, Changsha,410008, Hunan Province, China.; 4 Research Center of Carcinogenesis and Targeted Therapy, Xiangya Hospital, Central South University, Changsha, 410008, Hunan Province, China.; 5 Department of Breast and Thyroid Surgery, Tongji Medical College, Union Hospital, Huazhong University of Science and Technology, Wuhan, 430000, Hubei Province, China.; 6 Department of Gastrointestinal Surgery, the Sixth Affiliated Hospital, Sun Yat-sen University, Guangzhou, 510000, Guangdong Province, China.; 7 Department of Hepatobiliary Surgery, The First Affiliated Hospital of GuangXi Medical University, Nanning, 530021, Guangxi Province, China.; 8 International Joint Research Center of Minimally Invasive Endoscopic Technology Equipment & Standardization, Xiangya Hospital, Central South University, Changsha,410000, Hunan Province, China.

**Keywords:** FLOT1, gastric cancer, progression, metastasis, BCAR1

## Abstract

Flotillin-1 (FLOT1) is a member of the flotillin family and serves as a hallmark of lipid rafts involved in the process of signaling transduction and vesicular trafficking. Here, we find FLOT1 promotes gastric cancer cell progression and metastasis by interacting with BCAR1, through ERK signaling. FLOT1 regulates BCAR1 phosphorylation and translocation. Overexpression of FLOT1 increases, while knockdown of FLOT1 decreases gastric cancer cell proliferation, migration and invasion. BCAR1 knockdown could block FLOT1 induced gastric cancer cell proliferation, migration and invasion. Re-expression of wildtype rather than mutant BCAR1 (Y410F) could partially restore FLOT1 knockdown induced gastric cancer cell migration and invasion, while the restore could be inhibited by ERK inhibitor. Furthermore, FLOT1 and BCAR1 expression is closely related to gastric cancer patients' poor outcome. Thus, our findings confirm that BCAR1 mediates FLOT1 induced gastric cancer progression and metastasis through ERK signaling, which may provide a novel pathway for gastric cancer treatment.

## Introduction

According to GLOBOCAN 2020 estimated, gastric cancer was reported to be the fifth most commonly diagnosed cancer, and the third most common cause of cancer-related deaths globally, with 1,033,701 new cases of gastric cancer (representing 5.7% of all cancer cases diagnosed) and 769,000 deaths related to gastric cancer (representing 8.2% of all deaths from cancer) in the world in 2018^1^. The clinical symptoms of gastric cancer are dyspepsia, anorexia, early satiety, regurgitation, abdominal pain and weight loss^2^. However, the disease is often progressed into advanced stage even with distant metastasis once the symptoms are present when diagnosis, which imposes a huge burden on public health care. Therefore, further exploration in molecular mechanisms of gastric cancer progression and metastasis are urgently needed.

Flotillin-1 (FLOT1), member of the flotillin family (also known as the reggie family), is a hallmark of lipid rafts, who helps form a platform on plasma membrane or intracellular organelles for various molecules to conduct their functions^3^. FLOT1 plays roles in numerous biological processes including cancer-related processions^4^. Santamaría et al. reported that FLOT1 could induce prostate cancer cell proliferation by nuclear translocation and mitogenic activity^5^. Min Kang et al. showed that FLOT1 knockdown inhibited gastric cancer cell proliferation^6^. However, the molecular mechanism in it still needs to be investigated.

Breast Cancer Antiestrogen Resistance 1(BCAR1) also known as p130Cas, belongs to the Cas (Crk-associated substrate) family. BCAR1, serving as adaptor protein, is characterized by several protein-protein interaction domains that provide substrate binding sites, and multiple post-translational modification (mostly tyrosine and serine/threonine phosphorylation) that drives the process of a diverse spectrum of biological activities including gene transcription, immune response, cell adhesion, cell cycle progression, apoptosis, migration, and transformation^7^, ^8^. Recently, BCAR1 was also reported to be highly expressed and phosphorylated in many types of tumors, including melanoma, breast cancer, leukemias^9^-^11^.

In this study, we demonstrated that FLOT1 promotes gastric cancer cell proliferation, migration and invasion *in vitro* and *in vivo* through BCAR1/ERK signaling, and regulates BCAR1 phosphorylation and translocation, which may provide a novel therapeutic target for gastric cancer treatment.

## Materials and Methods

### Lentivirus construction and infection and plasmids transfection

The pLV19-FLOT1 lentivirus and plasmid contain the FLOT1 overexpression gene, HA tag and puromycin resistance gene, pLV19-BCAR1 lentivirus and plasmid contain the BCAR1 overexpression gene, Flag tag and puromycin resistance gene, the pDsRed2-N1-FLOT1 plasmid contains FLOT1 overexpression gene and red fluorescence protein (DsRed2), p-EGFP-C1-BCAR1 plasmid contains BCAR1 overexpression gene and enhanced green fluorescence protein (EGFP), FLOT1 shRNA (GCAGAGAAGTCCCAACTAATT, ATAGCTGAAGTTGCCTGAATG) lentivirus particle contains the puromycin resistance gene, BCAR1 shRNA (CCCAGGAATCTGTATATATTT, GCTGAAGCAGTTTGAACGACT) lentivirus particle contains the puromycin resistance gene. WT-BCAR1 plasmid contains wild type BCAR1 overexpression gene, and MT-BCAR1(T410F) plasmid contains a mutant BCAR1 overexpression gene, with the tyrosine in 410 site mutating into Phenylalanine. Lentivirus were infected into cells using polybrene(10μg/mL) and cultured with RPMI1640 medium containing puromycin(2μg/mL) for 14 days. Plasmids were transfected into cells using PEI.

### Cell culture

Human gastric cancer cell AGS and HGC-27 were purchased from Procell Life Science & Technology (Wuhan, China) and cultured in fetal bovine serum (Biological Industries, 04-001-1A) supplemented RPMI 1640 medium (Biological Industries, 01-100-1A) in cell incubator (37℃,5% CO2). All cell lines have been examined to exclude the mycoplasma contamination.

### Cell proliferation assay

Inoculated cells into 96-well plates one night before. Added 10μl MTT (3-(4,5)-dimethylthiahiazo (-z-y1)-3,5-di-phenytetrazoliumromide) solution into each well and incubated in the cell incubator, 4 hours later added 100μl DMSO into each well after MTT solution was removed. The absorbance of each well was measured at 570 and 630 nm with a Spectra Max M2 Microplate Reader (Molecular Devices, San Jose, CA, USA).

### Colony formation assay

Inoculated cells in six-well plate and incubated at 37 °C with 5% CO_2_ for 2-3 weeks. After cell colonies (about 50 cells) formed, washed each well twice using phosphate buffered saline (PBS). The plate was stained with 0.1% crystal violet (Beyotime, C0121) at 37 °C for 20 min after fixation with 4% Paraformaldehyde Fix Solution (Beyotime, P0099) for 20 min, and photographed after drying.

### Cell wound healing assay

Inoculated cells into a 12-well plate. Scratched the plate using 10μl pipettes after cell adherence, washed the plate with PBS buffer, and then cultured cells in RPMI1640 without serum. Observed wound closure at 0 and 24 or 48 hours (based on cell migration rate). Image J (1.4.3.67) was used to evaluate cell migration ability after the images were token in microscopic fields three times randomly (× horizon200) at both time point.

### Transwell invasion assay

Inoculated cells into the upper chamber of 8-μm-pore Transwells (BD Biosciences, San Jose, CA, USA) pre-coated with Matrigel, and the lower chamber was filled with RPMI1640 with 20% FBS to attract cells. Culture cells in cell incubator for two days. The 8-μm-hole was stained with 0.1% crystal violet for 30 min after fixation (4% paraformaldehyde), and three microscopic fields (× horizon 200) were selected randomly under the microscope, and then count cells.

### Colocalization analysis

293T cells or AGS cells were inoculated into a 24-well plate with a piece of 15mm microscope cover glass in each well. After cell adherence, transfected pDsRed2-N1-FLOT1, p-EGFP-C1-BCAR1 and the corresponding control plasmids into cells. 48 h later, stained with DAPI (Solarbio, C0065) for 3 min after fixation (4% paraformaldehyde). Imaging was observed with confocal laser microscope (Leica, Germany).

### Western blotting

Protein samples mixed with 5 × loading buffer were boiled at 100℃ for 5 minutes. Then cooled down the samples and subjected them into sodium dodecyl sulfate polyacrylamide gel, electrophoresed and transferred the sample to PVDF membrane. Then blocked the membrane with 5% skimmed milk for 1 hour, and then incubated the membrane with primary antibodies, at 4℃ overnight. Washed the membrane, incubated it into secondary antibodies, and washed it again for 3 times. At last, detected the signals with ChemiDoc (Bio-Rad, CA, USA). The primary antibodies used in this study were as follow: rabbit anti-FLOT1(Proteintech, 15571-1-AP, 1:1000), rabbit anti-ERK1/2 (Proteintech, 51068-1-AP, 1:1000), rabbit anti-p-ERK1/2 (Thr202/Tyr204) (Proteintech, 28733-1-AP, 1:1000), rabbit anti-p130 Cas (Cell Signaling Technology, #13846, 1:1000), rabbit anti-p-p130 Cas (Tyr410) (Cell Signaling Technology, #4011, 1:800), HA-Tag Rabbit mAb (Cell Signaling Technology, #3724, 1:1000), DYKDDDDK Tag Rabbit mAb(Cell Signaling Technology, #14793, 1:1000) antibodies.

### Immunoprecipitation

Proteins were extracted from 10 cm dishes of AGS or HGC-27 cells with full confluence. The whole protein lysates were immunoprecipitated with antibodies targeting against HA tag (CST, #3724 or Proteintech, 66006-2-Ig), DYKDDDDK tag (CST, #14793 or Proteintech, 66008-4-Ig) or IgG negative control (CST) at 4 °C overnight in the rotator, then added 50μL Protein A/G PLUS Agarose (Santa Cruz, sc-2003) beads into each tube and rotated for 2 h at 4 °C. Subsequently, wash the beads using PBST buffer 5minutes, 4 times. The immunoprecipitation complexes were separated from beads and analyzed using western blotting.

### *In vivo* xenograft mouse model and small animal PET/CT Imaging

The experiments were approved by the Research Ethics Committee of Xiangya Hospital, Central South University and IACUC of Hunan Yuanhe Biotechnology Co, Ltd for Animal Experiment (IACUC No.2022007). Male NOD-SCID mice aged four weeks were randomly divided into groups, five mice per group. For tumor growth assay, FLOT1 or BCAR1 knockdown (1×10^7^ cells) or overexpressed (5×10^6^ cells) AGS and HGC-27 cells were injected subcutaneously into the dorsal flank of mice. All the mice were monitored, and tumor size were measured every 3 (FLOT1 or BCAR1 OE and control group) or 6 (FLOT1-KD and control group) days (based on the rate of tumor growth). We used the formula V = (Length × Width^2^)/2 to calculate tumor volume. About 24 (FLOT1-OE and control group) or 36 (FLOT1-KD and control group) days later (based on the rate of tumor growth), mice were sacrificed, and tumor mass was collected and weighed. Immunohistochemistry and WB was performed on the mice tumor tissue to confirm the expression of FLOT1. Immunohistochemistry staining of Ki-67 was performed to examine cell proliferation rate.

For mice lung metastasis model, treated AGS cells (5×10^6^ cells) or treated HGC-27 cells (5×10^6^ cells) were injected into mice through tail vein. About 30 (FLOT1-OE and control group) or 60 (FLOT1-KD and control group) days later, mice were scanned using 18F-FDG PET-CT, and the uptake rate (tumor/muscle ratio) was compared. For imaging, all the mice fasted for at least 4 hours. PET/CT scan with nanoScan PET/CT scanner (Mediso, Hungary) was obtained after 50-60 min post-injection of 3.7-7.4 MBq 18F-FDG under anesthesia. The images were reconstructed, and regions of interest (ROIs) were drawn to obtain tumor-to-muscle ratios. The quantitative data were indicated as percentage injected dose per gram of tissue (% ID/g).

### Human GC tissue microarray preparation and Immunohistochemistry

We collected 102 pairs of tumors and the adjacent normal tissue of gastric cancer patients who underwent surgery at the department of general surgery, Xiangya hospital, Central South University. Based on voluntary principles, all patients were informed the purpose of this study, they could freely decide whether they want to participate in the investigation or not and gave their written informed consent before inclusion in this study. In the third year of follow-up survey, 80 out of 102 patients were followed up, the other 22 patients were lost to follow-up. The study was approved by the Research Ethics Committee of Xiangya Hospital, Central South University.

A gastric cancer tissue microarray containing 102 pairs of gastric cancer tissue and the adjacent normal tissue from samples of gastric patients was used. Paraffin sections were dewaxed in xylene for 15 minutes and hydrated using graded ethanol. After antigen heat repair using boiling water for 30 minutes, sections were treated with 3% hydrogen peroxide at room temperature for 10 minutes. After being blocked with 10% goat serum for 1 hour, the sections were incubated with rabbit anti-FLOT1 (Proteintech, 15571-1-AP, 1:200), rabbit anti-p130 Cas (Cell Signaling Technology, #13846, 1:200), rabbit anti-Ki67 (Proteintech, 27309-1-AP, 1:200), rabbit anti-p-ERK1/2 (Thr202/Tyr204) (Proteintech, 28733-1-AP, 1:200) antibodies at 4 °C overnight. Before being incubated with secondary antibodies for 1 hour, warm the sections at room temperature for 30 minutes. Staining the sections with DAB kit and hematoxylin. After hydrochloric acid differentiation, dehydration, transparency and sealing, the sections were examined and scored independently by three pathologists following the principle of blindness. The IHC scoring criterion was according to both the staining intensity and positive staining area. The staining intensity was graded as follows: 0, negative; 1, weak; 2, moderate; and 3, strong. The positive staining area was scored as follows: 1, 0-25%; 2, 26-50%; 3, 51-75%; and 4, >75%.

### Statistical analysis

Data were presented as the mean ± SD. Statistical analysis and visualization was conducted using SPSS 26.0 and GraphPad Prism V.8. The Student's t-test was used to analyze the two groups of data. Kaplan-Meier method was used to do Survival analysis, and comparisons were made using log-rank test. Correlations were analyzed using the Pearson correlation test based on IHC score. The statistical significance levels in this study are as follows: *<0.05, **<0.01, ***<0.001, and****<0.0001. The experiments were repeated three times.

## Results

### FLOT1 promotes gastric cancer cell proliferation, migration and invasion *in vitro*

**Figure 1:**
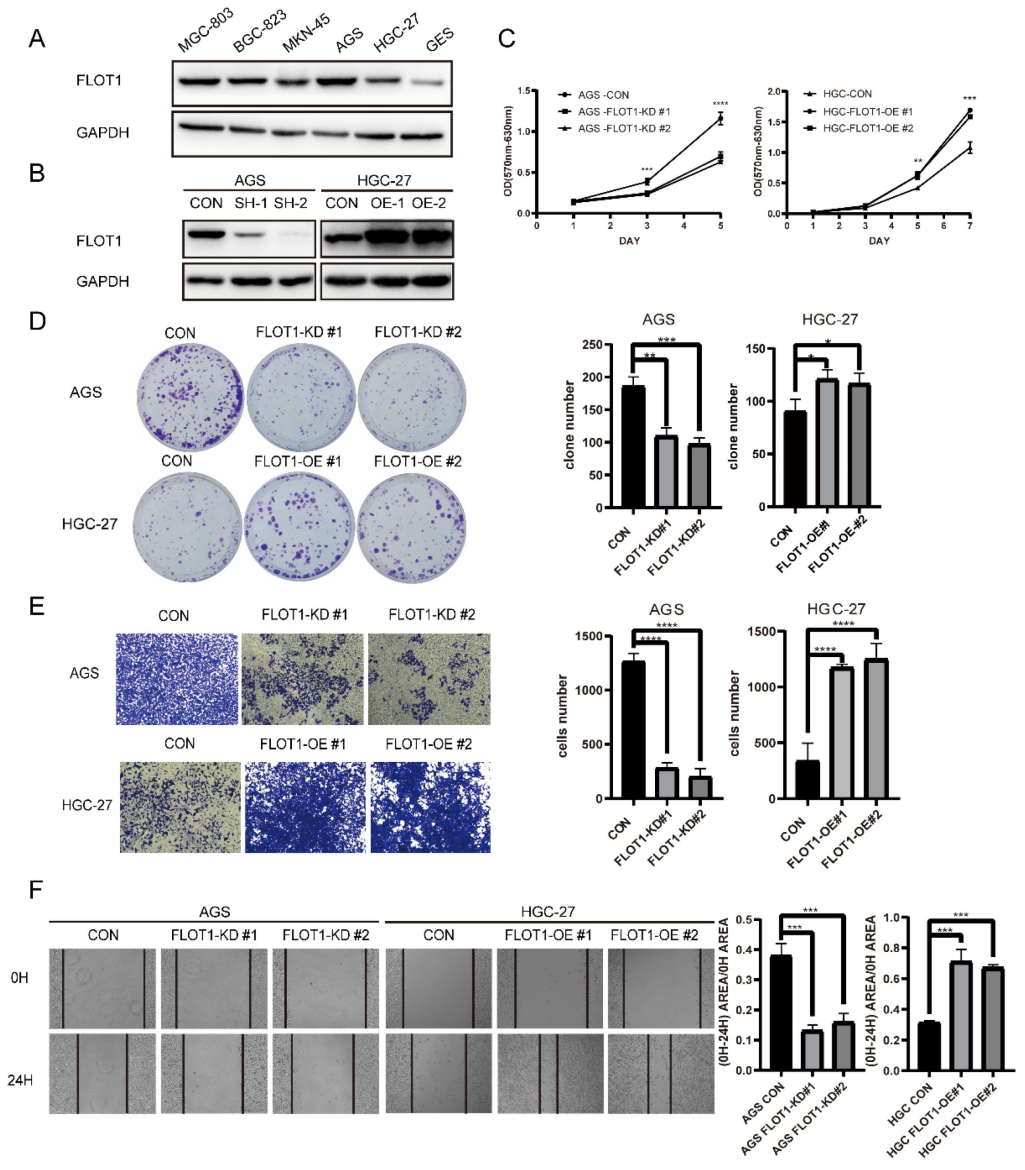
**FLOT1 promotes proliferation, migration and invasion in gastric cancer cells. A**. Western blotting for FLOT1 expression level in GES-1 and cultured GC cell lines. **B**. Western blotting for FLOT1 expression level in FLOT1-OE HGC-27 cell lines or FLOT1-KD AGS cell lines and the control cell lines. **C**. Cell proliferation assay for FLOT1-OE HGC-27 cell lines or FLOT1-KD AGS cell lines and the control cell lines. **D**. Colony formation assay and quantification data for FLOT1-OE HGC-27 cell lines or FLOT1-KD AGS cell lines and the control cell lines. **E**. Transwell invasion assay and quantification data for for FLOT1-OE HGC-27 cell lines or FLOT1-KD AGS cell lines and the control cell lines. **F**. Wound-healing assay and quantification data for FLOT1-OE HGC-27 cell lines or FLOT1-KD AGS cell lines and the control cell lines at 0 and 24h after wound scratch.

With the knowledge that FLOT1 was involved in several types of cancer and overexpressed in gastric cancer, we further investigated the function of FLOT1 in gastric cancer cells. Using western blotting, we examined the expression level of FLOT1 in a group of gastric cancer cell lines and normal gastric mucosa epithelial cell (GES-1) (Figure 1. A). We found that FLOT1 protein level was relatively high in AGS cell and low in HGC-27 cell. Then, we chose AGS cell to establish FLOT1 stable knockdown (FLOT1-KD) cell line and HGC-27cell was chosen for FLOT1 stable overexpression (FLOT1-OE) cell line establishment. The overexpression and knockdown of FLOT1 in gastric cancer cells were validated by western blotting (Figure 1. B). Using cell proliferation assay, colony formation assay, wound-healing assay and transwell invasion assay, we examined the proliferation, migration and invasion ability in FLOT1-OE HGC-27 cell, FLOT1-KD AGS cell and their corresponding control cells. Results showed that FLOT1 overexpression dramatically increased HGC-27 cell proliferation rate, migration and invasion ability. However, FLOT1 knockdown significantly decreased AGS cell proliferation rate, migration and invasion ability (Figure 1. C-F). With the results above, we concluded that FLOT1 promotes gastric cancer cell proliferation, migration and invasion.

### FLOT1 promotes tumorigenesis and metastasis in mouse model

**Figure 2:**
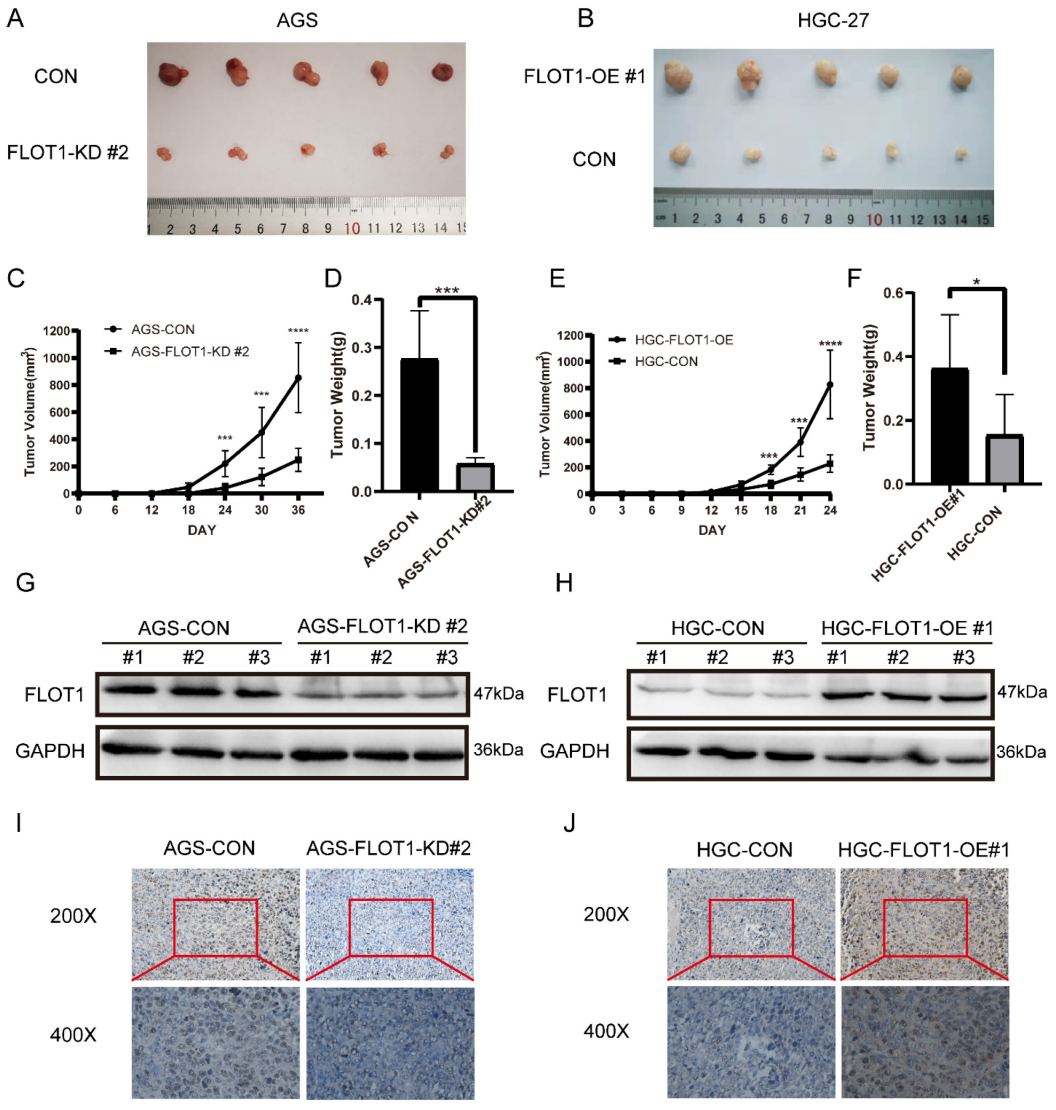
** FLOT1 promotes tumorigenesis in mouse model. A-J**. FLOT1-OE HGC-27 cells or FLOT1-KD AGS cells and the control cell lines were inoculated subcutaneously on the back of male NOD-SCID mice. (**C,E**) Tumors were measured and tumor volume were calculated on the indicated days. 36 or 24 days after inoculation, mice were sacrificed and tumors were collected (**A,B**) and weighted (**D,F**). The expression of FLOT1 in tumors was examined by western blotting (**G,H**). Ki-67 in mouse xenograft tumors were examined by Immunohistochemical staining (**I,J**).

To further investigate the role of FLOT1 in gastric cancer growth and development *in vivo*, FLOT1-OE HGC-27 cells (FLOT1-OE group) and FLOT1-KD AGS cells (FLOT1-KD group) and corresponding control cells were inoculated into NOD-SCID mice subcutaneously. Tumor size was measured, and tumor volume calculated (Figure 2. C, E). Mice were sacrificed when the length of the biggest tumor reached about 1.5cm, and tumors were collected and weighted (Figure 2. D, F). The overexpression and knockdown of FLOT1 in mice tumors were validated by western blotting (Figure 2. G, H). We found that, compared to the corresponding control group, mice inoculated with FLOT1-OE HGC-27 cells showed larger tumor (Figure 2. B), while the tumor size in FLOT1-KD group were much smaller (Figure 2. A). Moreover, the expression of Ki-67 in mice tumor were determined by immunohistochemistry (IHC) (Figure 2. I, J). Results also showed that Ki-67 expression level was higher in FLOT1-OE xenograft tumor and lower in FLOT1-KD group when compared to the corresponding control. These results indicated that the overexpression of FLOT1 increased tumor growth, while knockdown of FLOT1 decreased tumor growth remarkably in xenograft mouse model.

**Figure 3:**
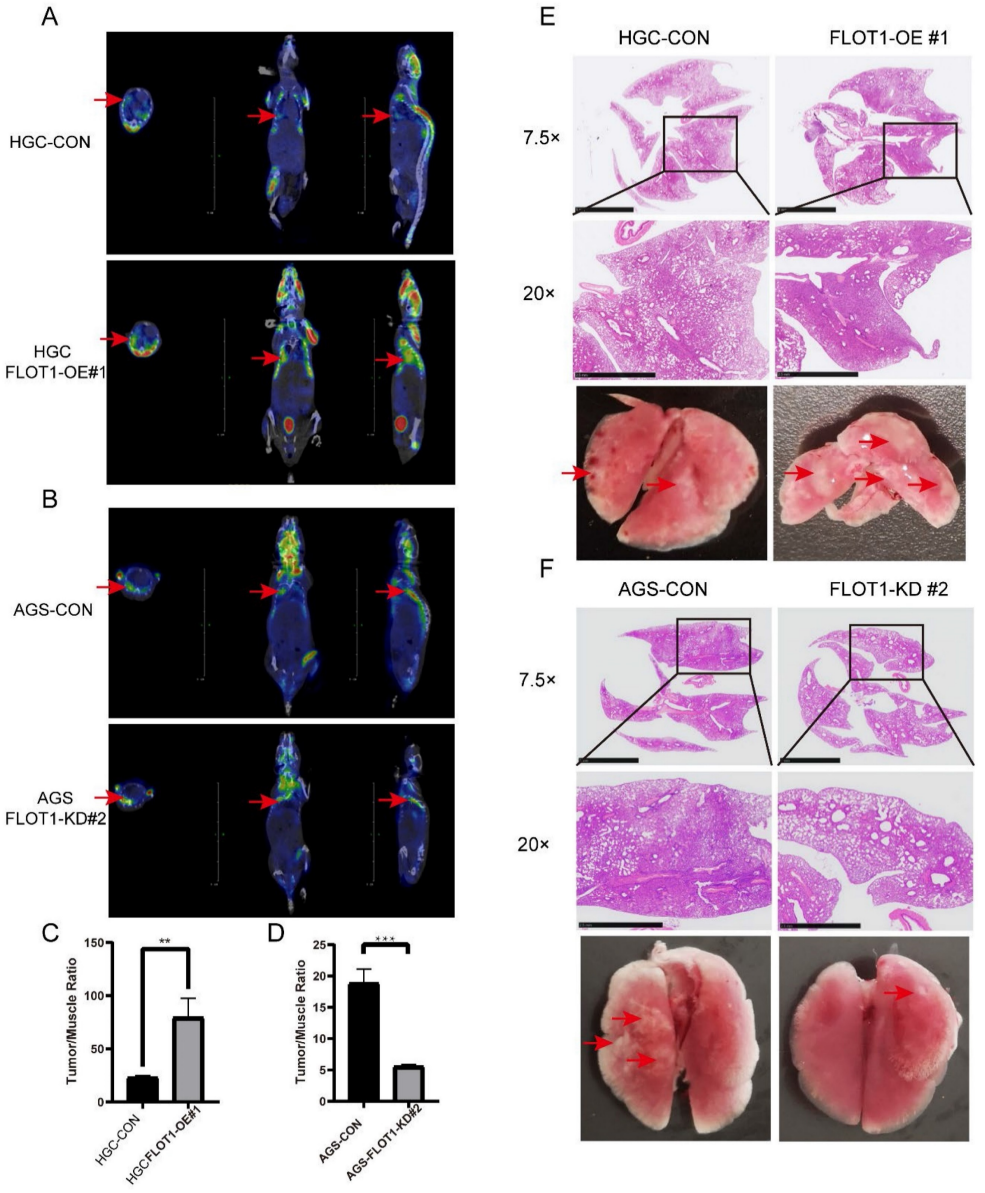
**FLOT1 promotes metastasis in mouse model. A-F.** FLOT1-OE HGC-27 cells or FLOT1-KD AGS cells and the control cell lines were injected into NOD-SCID mice through tail vein. 30(FLOT1-OE and control) or 60(FLOT1-KD and control) days later before sacrificed, mice were scanned by 18F-FDG PET-CT to check lung metastatic nodules (**A,B**) and the uptake rates of 18F-FDG (tumor/muscle ratio) were collected and compared (**C-D**). (**E-F**) Representative images of lungs and hematoxylin and eosin staining for paraffin-embedded lung sections. Arrows indicate lung metastatic nodules.

To determine the role of FLOT1 in metastasis in vivo, FLOT1-OE HGC-27 cells (FLOT1-OE group) and FLOT1-KD AGS cells (FLOT1-KD group) and corresponding control cells were injected into NOD-SCID mice intravenously through tail vein. 30 (FLOT1-OE and control group) or 60 (FLOT1-KD and control group) days later, we performed 18F-FDG PET-CT scan for mice in each group (Figure 3. A, B), and the uptake rate (tumor/muscle ratio) was compared. Then mice were sacrificed, and lungs were collected. In contrast to the control group, mice injected with FLOT1-OE HGC-27 cells exhibited more and larger lung metastatic nodules. Meanwhile, mice injected with FLOT1-KD AGS cells showed less and smaller metastatic nodules than the control group. The lung metastatic tumor nodules were confirmed by HE staining (Figure 3. E, F). Lung metastatic nodules in FLOT1-OE group showed higher tumor/muscle ratio, while the FLOT1-KD group showed lower tumor/muscle ratio (Figure 3. C, D), which indicated that FLOT1 increased gastric cancer metastasis *in vivo*.

### FLOT1 interacts with BCAR1 and regulates BCAR1 phosphorylation and translocation

**Figure 4:**
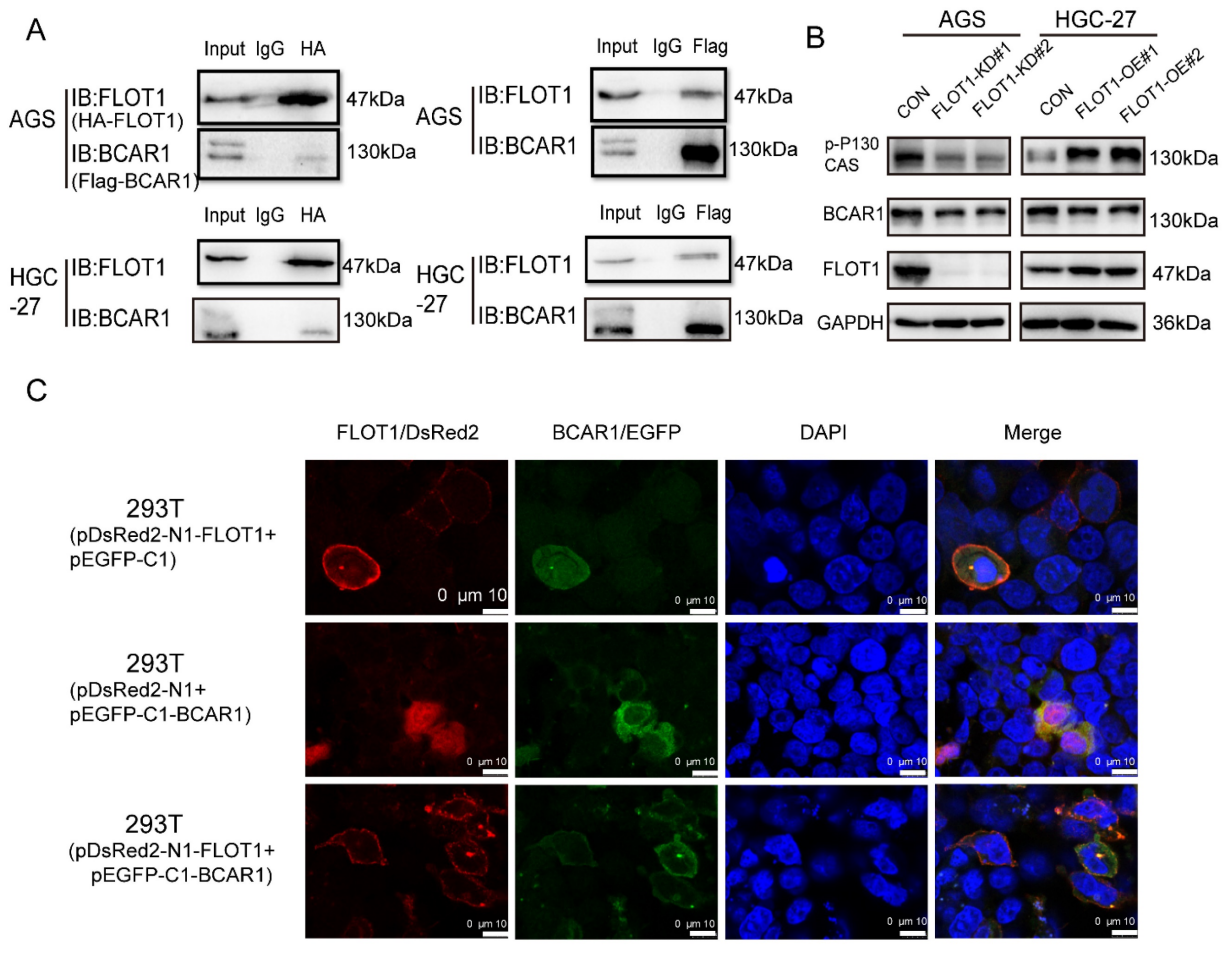
**FLOT1 interacts with BCAR1 and regulates BCAR1 phosphorylation and translocation. A**. FLOT1 co-immunoprecipitation with BCAR1(left) or BCAR1 co-immunoprecipitation with FLOT1(right) in AGS and HGC-27 cells. **B**. Western blotting for the expression of indicated proteins in FLOT1-OE HGC-27 cell lines or FLOT1-KD AGS cell lines and the control cell lines **C**. Confocal microscopy imaging analysis for the colocalization and translocation of exogenous pDsRed2-N1-FLOT1 and p-EGFP-C1-BCAR1 in 293T cell.

To further investigate the molecular mechanism of FLOT1 in gastric cancer, we transfected plasmids expressing HA-FLOT1 into AGS cells. 72h later cells were collected, protein was extracted, and immunoprecipitation (IP) was conducted by using anti-HA antibody. The complex of HA-FLOT1-IP protein was sent to proteomic analysis. The result suggested that BCAR1 is one of the proteins that interacts with FLOT1 (Supplementary Table 1). Then Co-IP was performed and the interaction between FLOT1 and BCAR1 in AGS and HGC-27 cells was confirmed (Figure 4. A). To investigate the location of FLOT1 and BCAR1, we transfected pDsRed2-N1-FLOT1 and p-EGFP-C1-BCAR1 plasmids alone or simultaneously into 293T cells and AGS cells. Confocal fluorescence microscopy imaging analysis showed that, FLOT1 was mainly localized at cell membrane, and BCAR1 was found in cytoplasm when pDsRed2-N1-FLOT1 and p-EGFP-C1-BCAR1 plasmid was transfected into cells alone. However, when pDsRed2-N1-FLOT1 and p-EGFP-C1-BCAR1 were co-transfected into 293T and AGS cells, BCAR1 re-localized from cytoplasm to membrane-associated places (Figure 4. C, Supplementary Figure 1).

It was reported that BCAR1 promotes cell migration in a manner dependent on the tyrosine phosphorylation of YXXP motifs in substrate binding domain^12^, so we examined the Tyr410 phosphorylation level of BCAR1, which was reported to be associated with cell migration^13^, in FLOT1-OE and FLOT1-KD gastric cancer cells and control cells using western blotting. The results showed that phosphorylated BCAR1, also named p-p130Cas (Tyr410) was significantly increased in FLOT1-OE cells and decreased in FLOT1-KD cells when compared with the corresponding control (Figure 4. B). These results strongly suggested that FLOT1 interacted with BCAR1 and regulated BCAR1 phosphorylation and translocation.

### BCAR1 plays oncogenic role in gastric cancer

**Figure 5:**
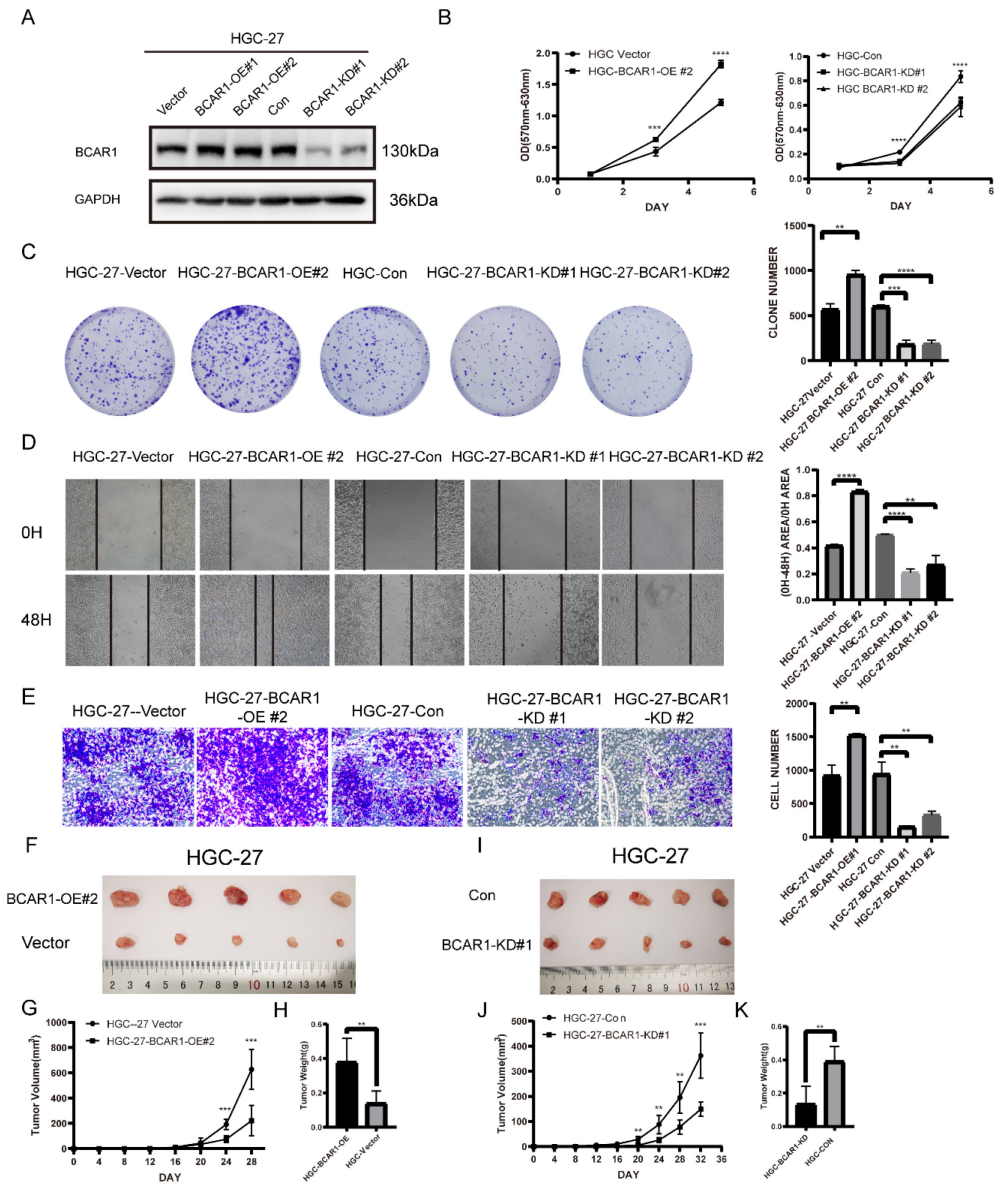
**BCAR1 plays oncogenic role in gastric cancer. A**. Western blotting for BCAR1 expression level in BCAR1-OE or KD HGC-27 cell lines and the control cell lines. **B**. Cell proliferation assay for the BCAR1-OE and control HGC cells (left) or BCAR1-KD and control HGC-27 cells(right). **C**. Colony formation assay and quantification data for BCAR1-OE or KD HGC-27 cell lines and the control cell lines. **D**. Wound-healing assay for BCAR1-OE or KD HGC-27 cell lines and the control cell lines at 0 and 48h after wound scratch. **E**. Transwell invasion assay for BCAR1-OE or KD HGC-27 cell lines and the control cell lines. **F-K**. BCAR1-OE or KD HGC-27 cell lines and the control cell lines were inoculated subcutaneously into NOD-SCID mice. (**G,J**) Tumors were measured and tumor volumes were calculated on the indicated days. 32 or 28 days after inoculation, mice were sacrificed and tumors were collected (**F,I**) and weighted (**H,K**).

To define the role of BCAR1 in gastric cancer, we established BCAR1 overexpressed and knockdown stable HGC-27cell lines and control cells by infecting BCAR1 OE and KD lentivirus and the corresponding control (Figure 5. A). We compared the cell proliferation, migration and invasion ability among these cells using cell proliferation assay, colony formation assay, wound-healing assay and transwell invasion assay in BCAR1 OE and KD HGC-27 cells and control cells. Results showed that BCAR1 overexpression significantly increased cell proliferation, migration and invasion ability. Meanwhile, knockdown of BCAR1 remarkably decreased the ability of cell proliferation, migration and invasion (Figure 5. B-E).

*In vivo*, we established xenograft mouse model by inoculating BCAR1 overexpressing (BCAR1 OE group), knockdown (BCAR1 KD group) HGC-27cells and the corresponding control cells into NOD-SCID mice subcutaneously. Mice were monitored until being sacrificed. The results showed that tumors in BCAR1 OE group were larger in size and weight than that in control group (Figure 5. F-H). While tumors in BCAR1 KD group were much smaller than control (Figure 5. I-K). These results indicated that BCAR1 plays an oncogenic role in gastric cancer *in vitro* and *in vivo*.

### BCAR1 mediates FLOT1 caused gastric cancer cell proliferation, migration and invasion through ERK signaling

**Figure 6:**
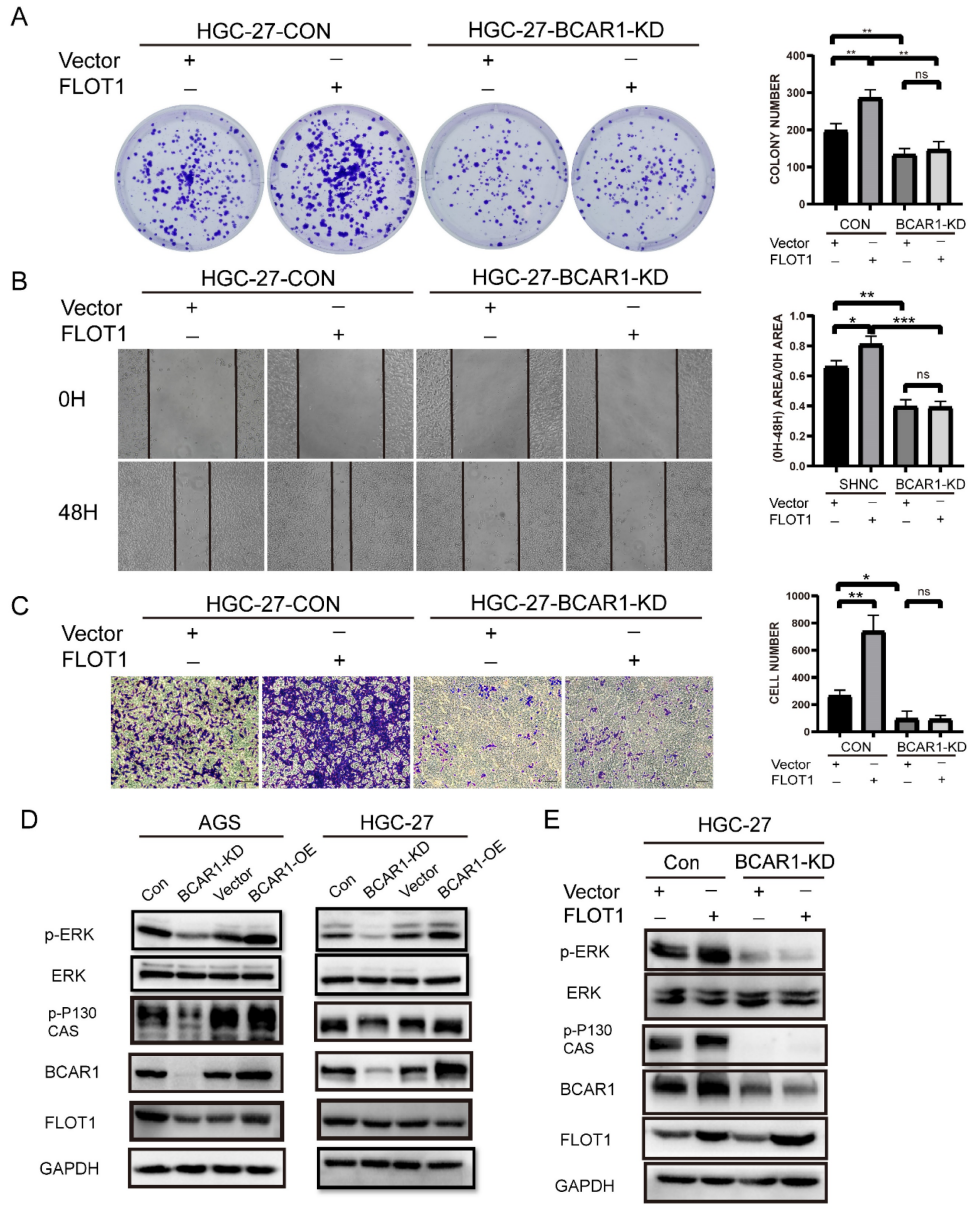
** BCAR1 mediates FLOT1 caused gastric cancer cell proliferation, migration and invasion through ERK signaling. A**. Colony formation assay and quantification data for BCAR1- KD and control HGC-27 cells transfected with FLOT1 overexpression or control plasmid. **B**. Wound-healing assay for BCAR1- KD and control HGC-27 cells transfected with FLOT1 overexpression or control plasmids at 0 and 48h after scratching. **C**. Trans-well invasion assay for BCAR1- KD and control HGC-27 cells transfected with FLOT1 overexpression or control plasmids. **D**. Western blotting for the expression of indicated proteins in BCAR1-OE or KD and the control cell lines in AGS (left) and HGC-27 (right) cell. **E**. Western blotting for the expression of indicated proteins in BCAR1- KD and control HGC-27 cells transfected with FLOT1 overexpression or control plasmid.

To investigate whether BCAR1 mediates FLOT1 induced gastric cancer cell proliferation, migration and invasion, we transfected FLOT1 OE and control plasmids into BCAR1 KD HGC-27 and the corresponding control cells. Then colony formation assay, cell wound healing assay and transwell assay were performed. Results showed that FLOT1 re-expression had minor effect on BCAR1-KD stable HGC-27 cells. That is to say, knockdown of BCAR1 blocked FLOT1 induced HGC-27 cell proliferation, migration and invasion (Figure 6. A-C).

By consulting references, we found that BCAR1 network could mediate biological signature through ERK, PAK and Hippo pathways^14^. Interestingly, FLOT1 also has been reported to be a very important regulator of classical MAP kinase signaling. Monia Amaddii and colleague^15^ demonstrated that, FLOT1 binds with three tiers of MAPK signaling simultaneously and could modulates the activation of ERK. Therefore, we tested p-ERK1/2 (Thr202/Tyr204) and ERK1/2 expression in BCAR1-OE and BCAR1-KD AGS and HGC-27cells, and corresponding control cells. Results showed that BCAR1 overexpression increased, while knockdown of BCAR1 decreased p-ERK1/2 (Thr202/Tyr204) expression significantly, compared with the control cells (Figure 6. D). Additionally, BCAR1 knockdown blocked FLOT1 induced ERK activation (Figure 6. E). These results suggested that BACR1 mediates FLOT1 induced gastric cancer cell proliferation, migration and invasion through ERK signaling.

### Phosphorylation of BCAR1 on Tyr410 is essential for FLOT1 induced GC cell migration and invasion

**Figure 7:**
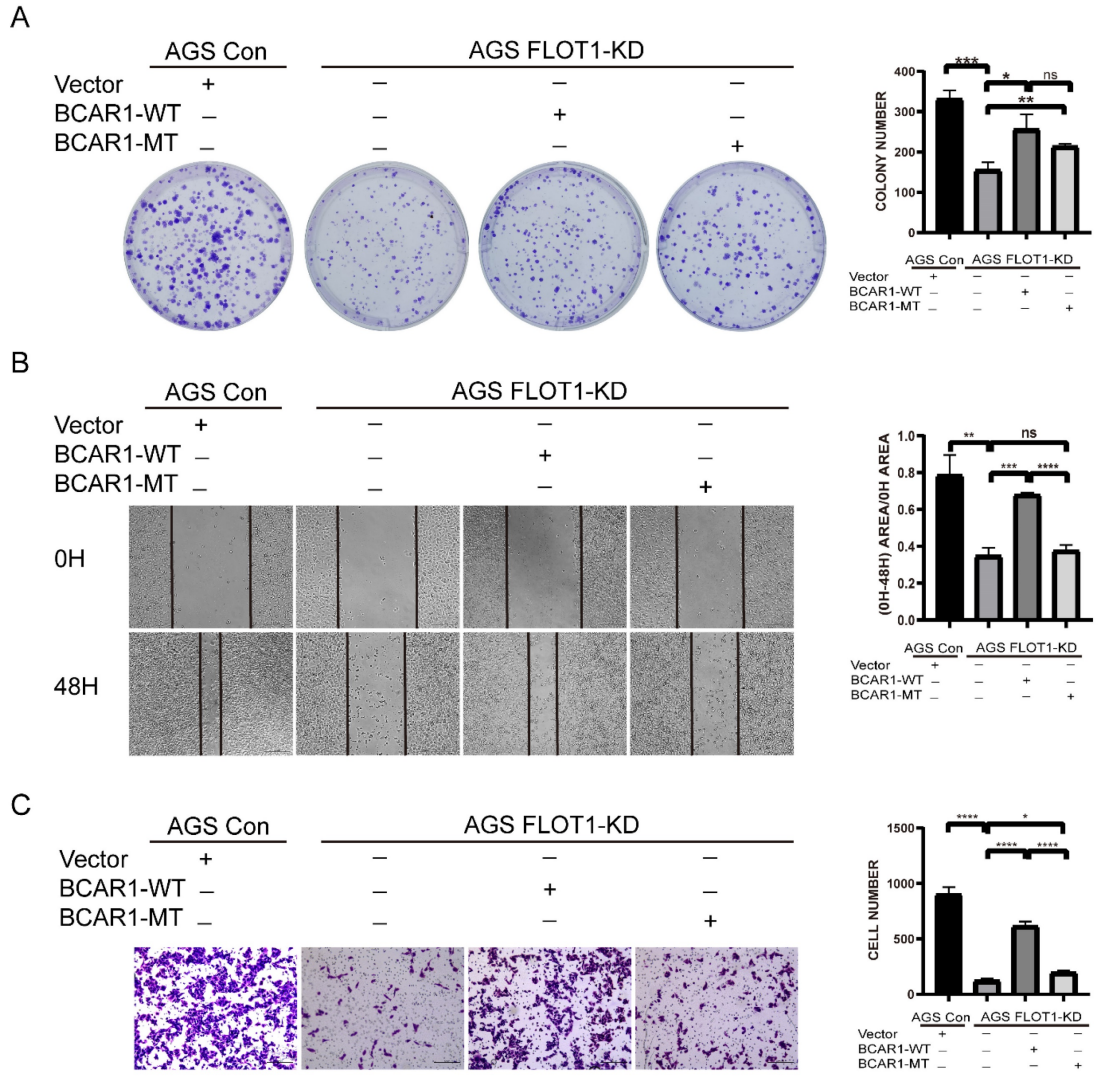
**Phosphorylation of BCAR1 on Tyr410 is essential for FLOT1 induced GC cell migration and invasion. A**. Colony formation assay and quantification data for FLOT1- KD AGS cell transfected with BCAR1-WT, BCAR1-MT or control plasmids and the control AGS cells. **B**. Wound-healing assay and quantification data for FLOT1- KD AGS cell transfected with BCAR1-WT, BCAR1-MT or control plasmids and the control AGS cells at 0 and 48h after scratching. **C**. Trans-well invasion assay for and quantification data for FLOT1- KD AGS cell transfected with BCAR1-WT, BCAR1-MT or control plasmids and the control AGS cells.

To investigate the effect of BCAR1 Tyr410 phosphorylation on FLOT1 induced gastric cancer cell proliferation, migration and invasion, we transfected WT-BCAR1 and MT-BCAR1 (Y410F) overexpression plasmids into FLOT1-KD stable AGS cell, and the abilities of cell proliferation, migration and invasion were examined. We found that overexpression of WT-BCAR1 could partially restore FLOT1 knockdown induced inhibition of gastric cancer cell proliferation, migration and invasion. However, MT-BCAR1(Y410F) could partially restored FLOT1 knockdown induced cell proliferation, not migration and invasion (Figure 7. A-C). Based on these results, it appears that phosphorylation of BCAR1 Tyr410 site is very important in FLOT1 induced gastric cancer cell migration and invasion.

Furthermore, we also used ERK inhibitor SCH772984 to treat WT-BCAR1 and MT-BCAR1 (Y410F) overexpressed FLOT1-KD AGS cells, and examined the ability of cell proliferation, migration and invasion. We observed that the restore of cell proliferation, migration and invasion because of WT-BCAR1 or MT-BCAR1 overexpression, was totally inhibited after ERK inhibitor administration (Supplementary Figure 3. A-F). These results further confirmed that ERK signaling mediated FLOT1/BACR1 induced gastric cancer cell proliferation, migration and invasion.

### FLOT1/BCAR1/ERK signaling is closely correlated with human gastric cancer

**Figure 8:**
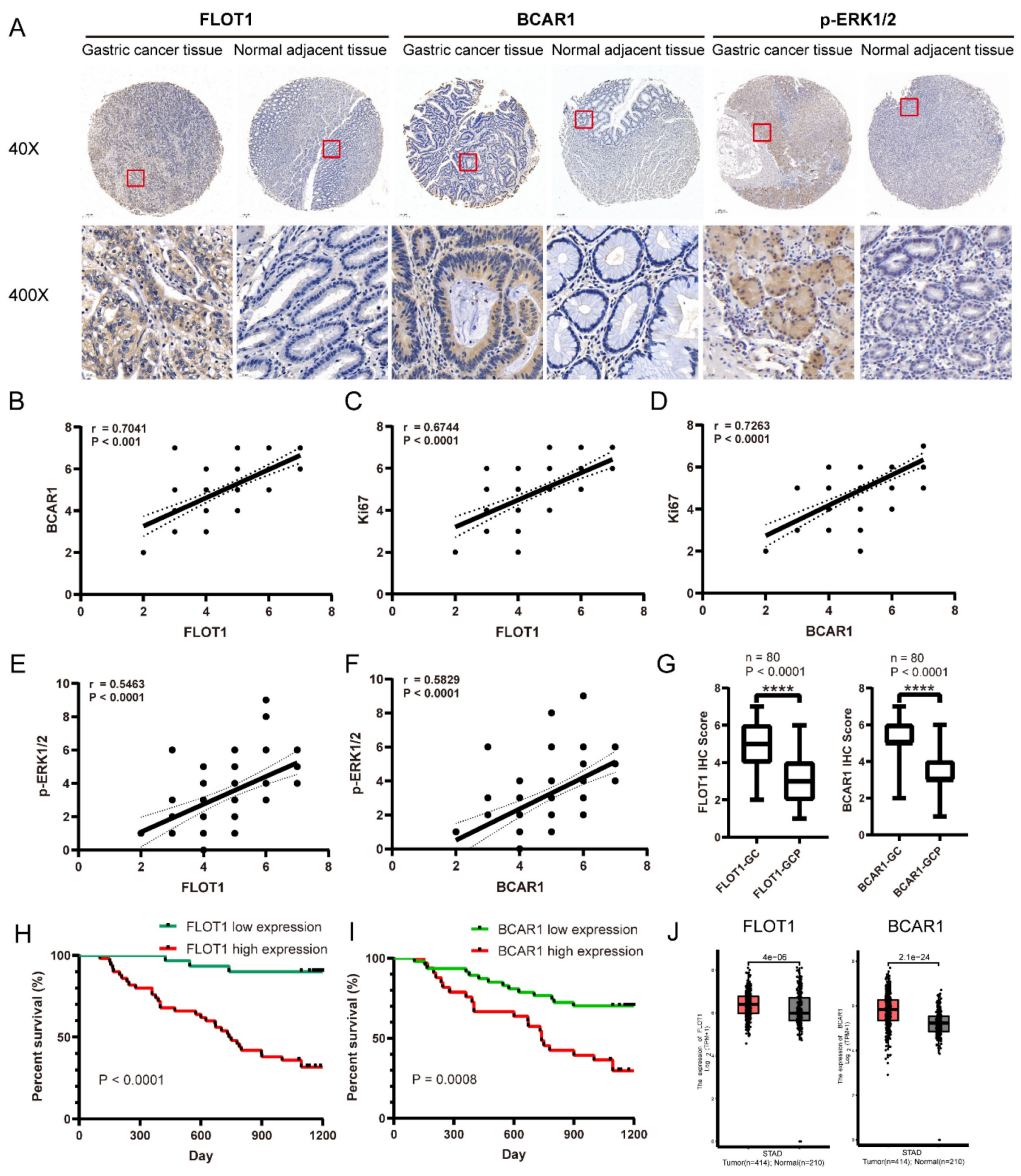
**FLOT1/BCAR1/ERK signaling is closely correlated with human gastric cancer. A**. Representative images of the IHC staining for FLOT1 and BCAR1 expression level in human gastric cancer tissues and paired adjacent normal gastric mucosa. **B**. The correlation between FLOT1 and BCAR1 expression in human gastric cancer tissues. **C.** The correlation between FLOT1 and Ki67 expression in human gastric cancer tissues. **D**. The correlation between BCAR1 and Ki67 expression in human gastric cancer tissues. **E**. The correlation between FLOT1 and p-ERK1/2 expression in human gastric cancer tissues. **F**. The correlation between BCAR1 and p-ERK1/2 expression in human gastric cancer tissues. Kaplan-Meier survival analysis showed the correlation between overall survival rate and FLOT1 expression. **G**. IHC staining score of FLOT1(left panel) and BCAR1(right panel) in gastric cancer tissues and adjacent normal mucosa (P < 0.0001). **H**. Kaplan-Meier survival analysis for the correlation of overall survival rate and FLOT1 expression. **I**. Kaplan-Meier survival analysis for the correlation of overall survival rate and BCAR1 expression. **J**. Comparison of FLOT1 mRNA expression in gastric cancer tissues (n = 414) and normal tissues (n = 210). Data from the TCGA (The Cancer Genome Atlas) (portal.gdc.cancer.gov/) database.

To determine the oncogenic role of FLOT1 and BCAR1 in gastric cancer patients, we analyzed the expression of FLOT1, BCAR1, p-ERK1/2 and Ki67 in 80 gastric cancer samples and the paired adjacent normal gastric mucosa using IHC staining. Results showed that the expression levels of FLOT1 and BCAR1 were significantly higher in gastric cancer tissue than the adjacent normal mucosa (Figure 8. A,G), and they were positively correlated with Ki67 and p-ERK1/2 expression (Figure 8. B-F). The association between the expression levels of FLOT1 and BCAR1 with clinical characteristics was analyzed and summarized. The expression levels of FLOT1 and BCAR1 were both significantly associated with tumor stage, depth of tumor invasion and regional lymph node (Table 1). Additionally, we found that FLOT1 and BCAR1 expression levels were negatively and significantly correlated with overall survival of gastric cancer patients during 3-year follow-up period (Figure 8. H,I). Data from TCGA also showed that FLOT1 and BCAR1 expression level was higher in gastric cancer tissue than the adjacent normal mucosa (Figure 8. J), which was highly coincide with our results, and the expression of FLOT1 and BCAR1 was significantly associated with progression free survival in gastric cancer patients in III-IV stages (Supplementary Figure 4. A,B). Additionally, FLOT1 expression was negatively correlated with overall survival in gastric cancer patients (Supplementary Figure 4. C). Collectively, all the results above indicated that FLOT1 and BCAR1 were overexpressed in gastric cancer tissue and was closely correlated with poor prognosis in gastric cancer patients.

## Discussion

With 1,033,701 new cases and 769,000 deaths according to data from GLOBOCAN 2020, gastric cancer was ranked the 5^th^ most diagnosed malignancy and the third most common cause of cancer related death worldwide^1^. Since there is no effective therapeutic option for gastric cancer patients in advanced stage, metastasis has been a major threat for gastric cancer patients. Therefore, a better knowledge of the molecular mechanism of gastric cancer progression and metastasis is in urgent need. In this study, we find FLOT1 promotes gastric cancer cell proliferation, migration and invasion through FLOT1/BCAR1/ERK pathway. FLOT1 interacts with BCAR1 and regulates the phosphorylation and translocation of BCAR1.

FLOT1, a member of the flotillin/reggie family, is located at the membrane compartments predominantly^16^. Flotillins assemble into large oligomers to form the micro domains called lipid rafts, on which small molecules interact with each other and through which signaling was transduced^17^-^19^. FLOT1 was also reported to be implicated in several types of cancer^6^, ^20^-^22^ and the expression level of FLOT1 affects its cellular distribution. When upregulated, FLOT1 accumulates in late endosomes in cancer^23^. In this work, we find overexpression of FLOT1 promotes proliferation, migration and invasion of gastric cancer cells and tumor growth, while knockdown of FLOT1 inhibits gastric cancer proliferation, migration and invasion in vitro and *in vivo*. Additionally, FLOT1 overexpressed in gastric cancer tissue when compared with the adjacent normal mucosal, and FLOT1 expression level is positively correlated with tumor stage, invasion, metastasis and patients' poor outcome.

FLOT1 implicated in signaling transduction, vesicle trafficking and cytoskeleton rearrangement by interacting with a variety of proteins, such as GPI-anchored proteins^22^, ^24^, several large transmembrane proteins^25^ and Src family kinases^26^-^28^. Accordingly, Jun Liu et al reported the interaction among adaptor protein CAP, Src family kinase fyn and FLOT1, suggesting the function of FLOT1 as a molecular link between complicated signaling complex and membrane lipid rafts^26^. In this study, we find a direct interaction between FLOT1 and BCAR1, another adaptor protein in Cas family. Furthermore, we found the expression level of FLOT1 was closely associated with phosphorylation of BCAR1 at Tyr 410, which plays crucial role in migration and chemoresistance of cancer therapy^13^.

BCAR1, also called p130Cas(Crk-associated substrate), was originally known for its closely association with Crk and crucial role in cell transformation by v-Src and v-Crk^29^. Structurally, BCAR1 is characterized by a substrate binding domain containing 15 repeats of YxxP sequence, in which the tyrosine residues can be phosphorylated by PTK and provide a binding site for SH2 or PTB, and a proline-rich region (RPLPSPP) and a tyrosine-containing sequence (YDYV) in the C-terminal domain, which binds to the Src SH3 and SH2 domain^30^. Due to these conserved motifs, BCAR1 is highly phosphorylated and promotes protein-protein interaction^7^, ^31^-^34^. And once phosphorylated, BCAR1 may relocalized from cytoplasm to membrane-associated cell fraction^35^, which coincides well with our observation from confocal. We also identified the oncogenic role of BCAR1 in gastric cancer *in vitro* and *in vivo*. Overexpression of BCAR1 promotes gastric cancer cell proliferation, migration, invasion and tumor growth. While knockdown of BCAR1 inhibits the ability of cell proliferation, migration, invasion and tumor growth in gastric cancer. Additionally, the phosphorylation of BCAR1 at Tyr 410 is specifically associated with FLOT1 induced gastric cancer invasion and migration.

ERK signaling, as a classical oncogenic pathway, has been reported to transduce both FLOT1 and BCAR1 induced tumorigenesis^15^, ^36^, ^37^. In our study, we also observed the activation of ERK increased in both FLOT1 or BCAR1 overexpressed HGC-27cells. However, when we knockdown BCAR1 in FLOT1 overexpressed HGC-27cell, we observed the inhibition of ERK activation, and so was the ability of cell proliferation, migration and invasion. Moreover, BCAR1 overexpression can partially restore FLOT1 knockdown induced gastric cancer cell proliferation, migration and invasion. But the recovery can be inhibited by ERK inhibitor. All these results from rescue experiments suggested that BCAR1 mediate FLOT1 induced gastric cancer proliferation, migration and invasion through ERK signaling.

However, FLOT1 has not been reported to have protein kinases activity. Then how was BCAR1 phosphorylated in gastric cancer cells in which FLOT1 was upregulated? By consulting references, we find that BCAR1 was an important substrate of Src, and Src phosphorylates at least ten tyrosine residues located at SH2-and-PTB-binding domain of BCAR1 between Tyr238 and Tyr 414, which are closely associated with cell migration^12^, ^38^. Therefore, Src may have vital role in the regulation of FLOT1 on BCAR1 phosphorylation, which needs to be further verified and offers a good instruction for our further research. Furthermore, the mechanism in metastatic tumor is never limited to tumor cells due to the existence of intratumoral heterogeneity, complex tumor microenvironment, and immunosuppressive tumor ecosystem in metastatic cancer^39^. To completely unveil the mechanism of FLOT1 in gastric cancer progression and metastasis, we should not only focus on tumor cell itself and the function of FLOT1, but also other cell types, small molecular and the whole tumor ecosystem comprehensively and multifacetedly^40^, ^41^.

Taken together, our study indicates that, BCAR1 mediates FLOT1 induced gastric cancer progression and metastasis through ERK signaling, which may present a novel therapeutic approach for gastric cancer treatment.

## Supplementary Material

Supplementary figures and table.

## Figures and Tables

**Table 1:** Correlation between the expression of FLOT1, BCAR1 and clinicopathological characteristics in 80 cases of human gastric cancer tissue

		FLOT1			BCAR1		
Characteristic	n	Low	High	P	Low	High	P
**Age (years)**							
≤ 60	40	15	25	0.2637	11	29	0.6197
> 60	40	11	29		8	32	
**Gender**							
Male	51	15	36	0.5656	12	39	0.6914
Female	29	12	17		7	22	
**Tumor stage**							
I-II	24	8	16	0.0124	8	16	0.0487
III-IV	56	19	37		11	45	
**Depth of tumor invasion**							
T1-T2	10	8	2	0.0000	8	2	0.0002
T3-T4	70	19	51		11	59	
**Regional lymph node invasion**							
Yes	63	18	45	0.0051	12	51	0.0152
No	17	9	8		7	10	
**Regional nerve invasion**							
Yes	47	11	36	0.0105	8	39	0.0932
No	33	16	17		11	22	
**Regional vascular invasion**							
Yes	45	11	34	0.0362	7	38	0.0810
No	35	16	19		12	23	
**Differential degree**							
Well and moderate	29	12	17	0.6721	10	19	0.2455
Poor and undifferentiated	51	15	36		9	42	

*P < 0.05* was considered to be statistically significant
