# Supplementary material for: FLOT1 promotes gastric cancer progression and metastasis through BCAR1/ERK signaling
Source: Int J Biol Sci. 2023 Oct 2;19(16):5104–19. doi: 10.7150/ijbs.82606 (PMC10620819; doi:10.7150/ijbs.82606)
Supplement: Supplementary file 1 — Supplementary figures and table. [file ijbsv19p5104s1.pdf]

Supplementary Figure 1.

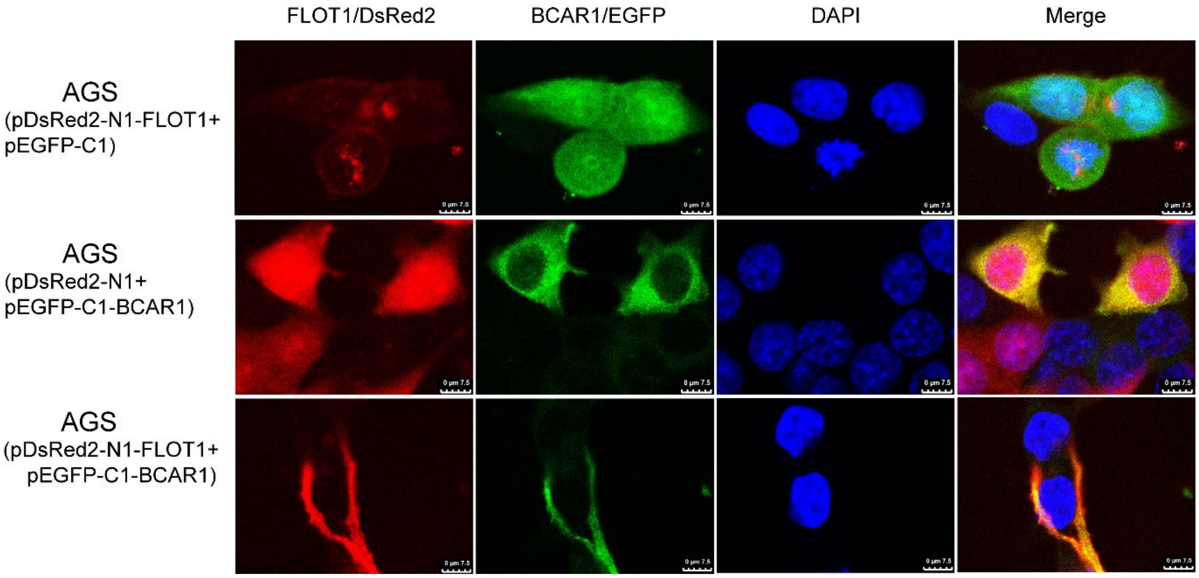

Supplementary Figure 1. Confocal microscopy imaging analysis for the colocalization and translocation of exogenous pDsRed2-N1-FLOT1 and p-EGFP-C1-BCAR1 in AGS cell.

Supplementary Figure 2.

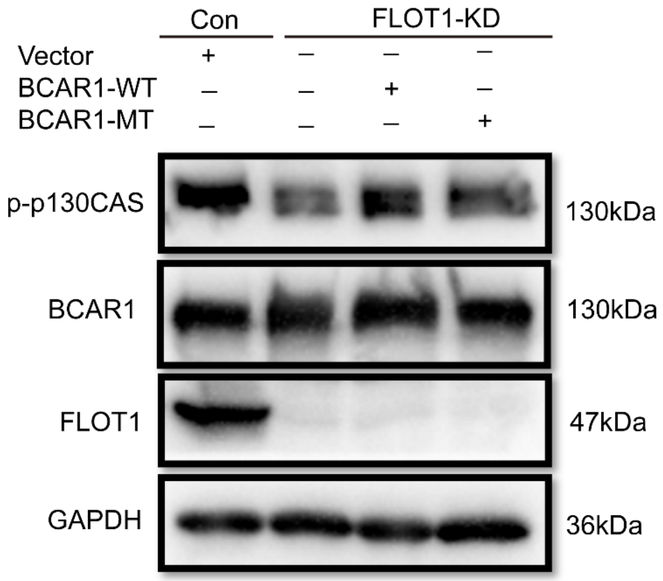

Supplementary Figure 2. Western blotting for the expression of indicated proteins in FLOT1- KD AGS cell transfected with BCAR1-WT, BCAR1-MT or control plasmid and the control AGS cells.

Supplementary Figure 3.

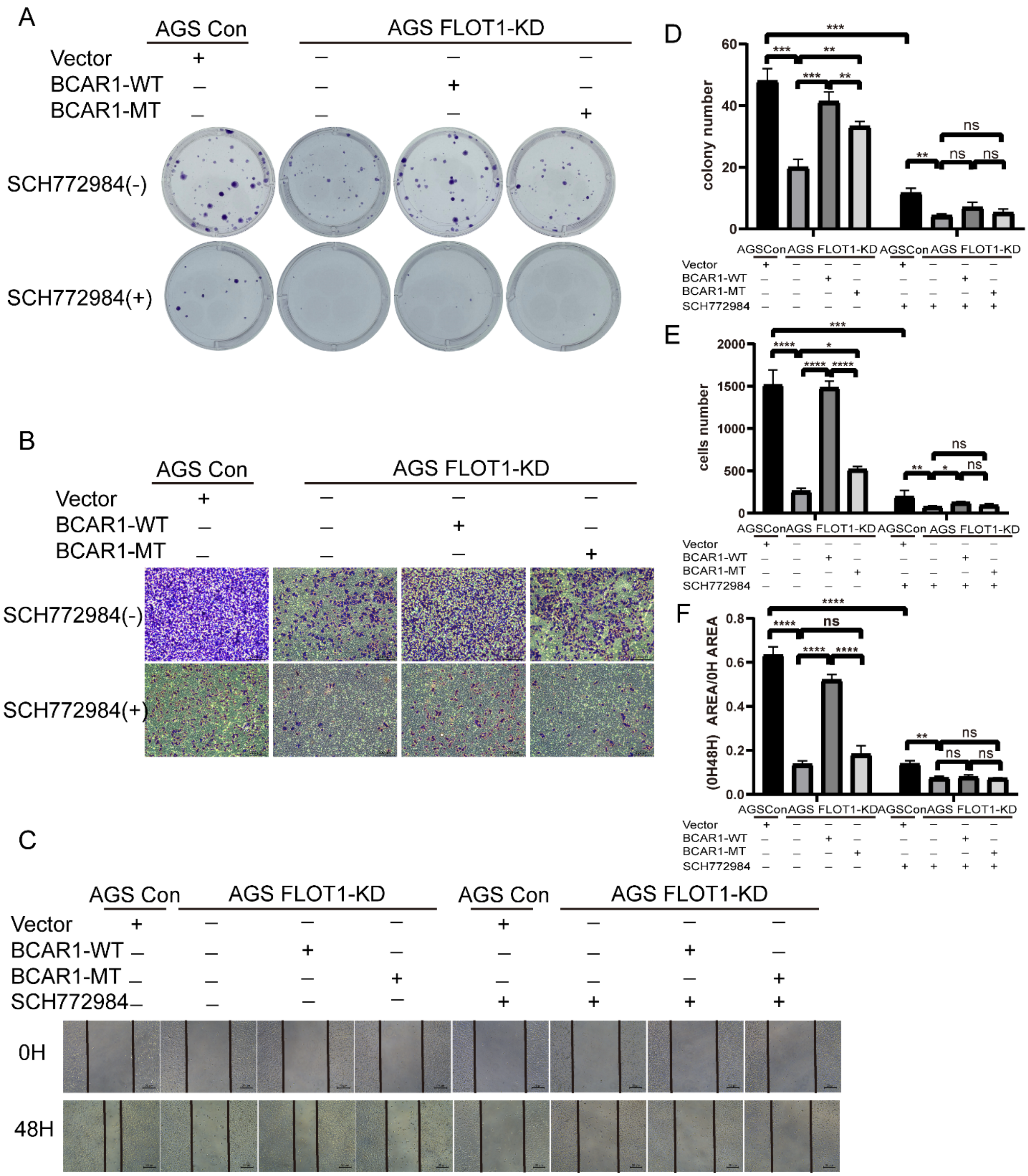

Supplementary Figure 3. Function experiment for ERK inhibitor treated FLOT1- KD AGS cell transfected with BCAR1-WT, BCAR1-MT or control plasmid and the control AGS cells.

A. Colony formation assay and quantification data(**D**) for ERK inhibitor treated FLOT1- KD AGS cell transfected with BCAR1-WT, BCAR1-MT or control plasmid and the control AGS cells. **B**. Wound-healing assay and quantification data(**E**) for ERK inhibitor treated FLOT1- KD AGS cell transfected with BCAR1-WT, BCAR1-MT or control plasmid and the control AGS cells at 0 and 48h after scratching. **C**. Trans-well invasion assay for and quantification data(**F**) for ERK inhibitor treated FLOT1- KD AGS cell transfected with BCAR1-WT, BCAR1-MT or control plasmid and the control AGS cells.

Supplementary Figure 4.

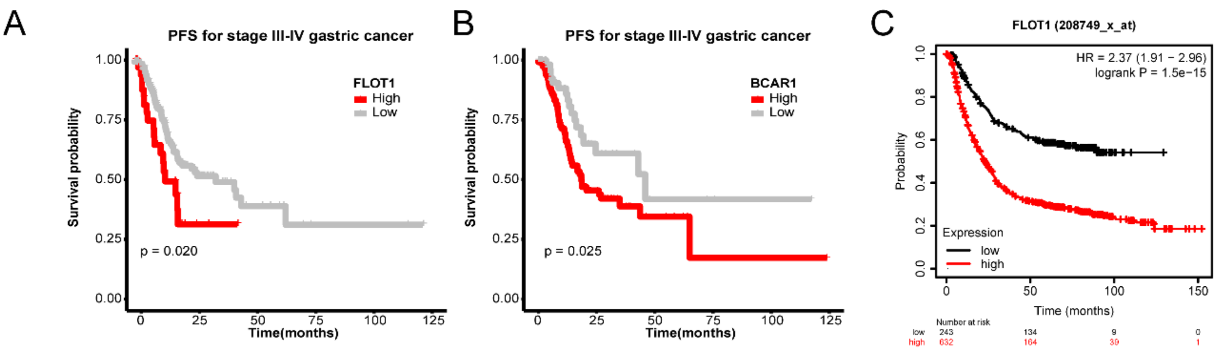

Supplementary Figure 4. A. The correlation of FLOT1 expression with progression-free survival in patient belonging to stage III+IV. Data from the TCGA(The Cancer Genome Atlas)(portal.gdc.cancer.gov/) database. **B**. The correlation of BCAR1 expression with progression-free survival in patient belonging to stage III+IV. Data from the TCGA(The Cancer Genome Atlas) (portal.gdc.cancer.gov/)database. **C**. The correlation of FLOT1 expression with patient overall survival. Data from the Kaplan–Meier Plotter (http://kmplot.com) database (FLOT1 (208749_x_at)).

Supplementary table:

Supplementary Table 1. Proteins interact with FLOT1 revealed in proteinomic analysis

No.	Accession	Protein names	Gene names	MW [kDa]	Protein score
1	P04264	Keratin, type II cytoskeletal 1	KRT1	66.00	2407.20
2	P13645	Keratin, type I cytoskeletal 10	KRT10	58.79	1652.63
3	P35527	Keratin, type I cytoskeletal 9	KRT9	62.03	1587.28
4	P35908	Keratin, type II cytoskeletal 2 epidermal	KRT2	65.39	1002.88
5	P68871	Hemoglobin subunit beta	HBB	15.99	608.49
6	Q86YZ3	Hornerin	HRNR	282.23	506.35
7	O75955	Flotillin-1	FLOT1	47.33	503.91
8	P02538	Keratin, type II cytoskeletal 6A	KRT6A	60.01	501.44
9	P02533	Keratin, type I cytoskeletal 14	KRT14	51.53	435.72
10	P02768	Albumin	ALB	69.32	367.82
11	P13647	Keratin, type II cytoskeletal 5	KRT5	62.34	331.12
12	P08779	Keratin, type I cytoskeletal 16	KRT16	51.24	330.34
13	P69905	Hemoglobin subunit alpha	HBA1	15.25	293.79
14	Q04695	Keratin, type I cytoskeletal 17	KRT17	48.08	246.16
15	P09211	Glutathione S-transferase P	GSTP1	23.34	156.51
16	P62269	40S ribosomal protein S18	RPS18	17.71	132.42
17	P38646	Stress-70 protein, mitochondrial	HSPA9	73.64	114.26
18	P0DOX7	Immunoglobulin kappa light chain	---	23.36	69.15
19	P68363	Tubulin alpha-1B chain	TUBA1B	50.12	59.68
20	P25705	ATP synthase subunit alpha, mitochondrial	ATP5F1A	59.71	55.27
21	Q02413	Desmoglein-1	DSG1	113.68	51.55
22	P13804	Electron transfer flavoprotein subunit alpha, mitochondrial	ETFA	35.06	50.50
23	A5A3E0	POTE ankyrin domain family member F	POTEF	121.37	50.03
24	P36957	Dihydrolipoyllysine-residue succinyltransferase component of 2-oxoglutarate dehydrogenase complex, mitochondrial	DLST	48.72	49.23
25	Q8TB92	3-hydroxy-3-methylglutaryl-CoA lyase, cytoplasmic	HMGCLL1	39.49	45.20
26	P56945	Breast cancer anti-estrogen resistance protein 1	BCAR1	93.31	42.32
27	P62249	40S ribosomal protein S16	RPS16	16.44	41.66
28	P0DOX8	Immunoglobulin lambda-1 light chain	---	22.82	40.43
29	P11142	Heat shock cognate 71 kDa protein	HSPA8	70.85	40.14
30	P14923	Junction plakoglobin	JUP	81.69	38.26
31	P15924	Desmoplakin	DSP	331.57	37.26

32	Q96HU8	GTP-binding protein Di-Ras2	DIRAS2	22.47	37.02
33	O76031	ATP-dependent Clp protease ATP-binding subunit clpX-like, mitochondrial	CLPX	69.18	32.01
34	A0A087WW87	Immunoglobulin kappa variable 2-40	IGKV2-40	13.30	29.75
35	P0DOX5	Immunoglobulin gamma-1 heavy chain	---	49.30	29.56
36	Q5D862	Filaggrin-2	FLG2	247.93	28.33
37	P33992	DNA replication licensing factor MCM5	MCM5	82.23	26.73
38	Q5T749	Keratinocyte proline-rich protein	KPRP	64.09	0.00
